# Model-driven engineering for digital twins: a graph model-based patient simulation application

**DOI:** 10.3389/fphys.2024.1424931

**Published:** 2024-08-12

**Authors:** William Trevena, Xiang Zhong, Amos Lal, Lucrezia Rovati, Edin Cubro, Yue Dong, Phillip Schulte, Ognjen Gajic

**Affiliations:** ^1^ Department of Industrial and Systems Engineering, University of Florida, Gainesville, FL, United States; ^2^ Mayo Clinic, Rochester, MN, United States

**Keywords:** digital twin, virtual patient simulation, graph model, full-stack application architecture, critical care

## Abstract

**Introduction:**

Digital twins of patients are virtual models that can create a digital patient replica to test clinical interventions *in silico* without exposing real patients to risk. With the increasing availability of electronic health records and sensor-derived patient data, digital twins offer significant potential for applications in the healthcare sector.

**Methods:**

This article presents a scalable full-stack architecture for a patient simulation application driven by graph-based models. This patient simulation application enables medical practitioners and trainees to simulate the trajectory of critically ill patients with sepsis. Directed acyclic graphs are utilized to model the complex underlying causal pathways that focus on the physiological interactions and medication effects relevant to the first 6 h of critical illness. To realize the sepsis patient simulation at scale, we propose an application architecture with three core components, a cross-platform frontend application that clinicians and trainees use to run the simulation, a simulation engine hosted in the cloud on a serverless function that performs all of the computations, and a graph database that hosts the graph model utilized by the simulation engine to determine the progression of each simulation.

**Results:**

A short case study is presented to demonstrate the viability of the proposed simulation architecture.

**Discussion:**

The proposed patient simulation application could help train future generations of healthcare professionals and could be used to facilitate clinicians’ bedside decision-making.

## 1 Introduction

Digital twins are virtual representations of systems that interact with the physical system bi-directionally (Lal et al., 2020a). With the increasing availability of electronic health records and sensor-derived patient data, digital twins hold significant potential in the healthcare sector. In particular, digital twin technology enables the creation of computerized replicas of patients, allowing simulation of diverse clinical scenarios and testing of interventions *in silico* without subjecting real patients to avoidable risk.

A virtual patient is a digital model able to be identified from relevant bedside data and provides prediction in response to modeled inputs. Previous works have demonstrated that virtual patient simulations can be successfully utilized to train medical professionals across an array of specialties (Kononowicz et al., 2019; Lee et al., 2020; Lee and Lee, 2021; Wu et al., 2022). However, many of the previously introduced virtual patient simulation models progress only along a limited number of hand-crafted or predetermined pathways, such as looped, serious branch games, and linear text-based scenarios (Berger et al., 2018). Other examples include virtual patient simulations that progress along decision trees (Hwang et al., 2022), and another recent work (Goldsworthy et al., 2022) utilized a commercial virtual patient simulation application, First2Act, which supports only seven simulation scenarios. Although such simulation architectures have been effectively utilized to train medical professionals, they are hard to scale as each new scenario must be crafted by hand.

Recently, computational simulation models have been proposed, which seek to dynamically model the evolution of organ systems within the human body. One such simulation focused specifically on modeling how the cardiovascular system evolves based on a set of time-varying, simultaneous differential equations (Burkhoff and Dickstein, 2024). Another example is glycemic control, and there have been multiple metabolic system models based on decades of research (Chu et al., 2023). Glycemic control protocols have been optimized using these models. In addition, virtual patient models to predict lung mechanics evolution with changing ventilator settings (mechanic ventilator models) are critical to effectively managing acute respiratory symptoms for critically ill patients, but the scope of the models is very limited (Zhou et al., 2021). These models focus primarily on the one organ system and are developed based on medical, physiological, or biological knowledge, i.e., physics-based models.

In summary, digital twin applications on virtual patient modeling have gained success in modeling individual organs for drug discovery and precision medicine (Venkatesh et al., 2022; Moingeon et al., 2023), but these models rely on the full characterization of the biological and physiological functions at the cell level or the organ level. From bench to bedside, it is important to understand how the organ systems interact and orchestrate the patient’s health. For critically ill patients, the capability of modeling and predicting patient trajectories under different treatment regimens would greatly support clinical decision-making, improving patient safety and health outcomes. However, our current knowledge about the human body does not allow us to accurately depict all organ system functions using physical or mechanical models (Rovati et al., 2024). There have been emerging efforts to develop patient or human digital twins based on predictive modeling using AI and machine learning (Vallée, 2023; Katsoulakis et al., 2024; Laubenbacher et al., 2024). Despite having superior predictive capacity, the interpretability of these models is typically limited. Meanwhile, graphical models of the biomarkers of each major organ system would allow us to encode essential interactions among these biomarkers and allow for good interpretability for educational purposes and practical clinical bedside use.

Alternatively, our preliminary work (Trevena et al., 2022) proposes a virtual patient simulation architecture driven by graph-based models and focuses on patient-level simulation, i.e., modeling of the evolution of the virtual patient, determined by directed acyclic graphs (DAGs) depicting the complex pathophysiological interactions that occur within the human body. This graph-based modeling provides a more accurate and transparent presentation of complex relationships between multiple variables in a complex adaptive system where the data is often characterized by intricate interdependence and association. The improved transparency and interoperability in return ensures that the underlying expert rules building upon which the DAGs are crafted can be validated using patient data. It also allows for better visualization of variable relationships and the reasoning behind the model’s decision output. The modular and flexible nature of the graph-based model also provides an opportunity to independently and iterative refine different organ systems (respiratory, cardiovascular, neurological, etc.) as discrete models to improve efficiency, and to create a more streamlined approach to incorporate new knowledge in a specific organ system without overhauling the entire model.

The goal of this research is to develop a new highly scalable full-stack architecture for a cross-platform patient simulation application driven by graph-based models, and to present a proof-of-concept of the proposed architecture to illustrate its viability. To realize the graph-based virtual patient simulation at scale, we prioritize a highly reliable, fault-tolerant, and maintainable architecture. As we aim to develop the application as a bedside decision-support tool for clinicians in actual clinical settings, the application needs to adapt swiftly and efficiently to fluctuating user demand, and to accommodate a wide range of user devices including laptops, tablets, and smartphones with diverse operating systems (iOS, Android, etc.). Our proposed architectural approach addresses these needs in an integrated manner, contributing a sustainable and practical solution to the field. Specifically, the architecture comprises three core components: a cross-platform front-end application that clinicians and trainees use to run the simulation, a cloud-hosted simulation engine that performs all the necessary computations for each user’s simulation, and a graph database that hosts the graph model used by the simulation engine to drive each simulation. By integrating these elements, we present a highly-scalable full-stack simulation application architecture, which effectively addresses the identified challenges and paves the way for a new paradigm in patient simulation and dynamic system simulation based on graph models. Although the application focus of this paper is on modeling a virtual patient, the architecture presented in this paper could be adapted to support other dynamic systems such as mechanical, physical, and physiological systems that are graph-based, e.g., Sanchez-Gonzalez et al. (2018); Tu et al. (2019); Yang et al. (2021).

In the following sections of this paper, we elaborate on how the components of our proposed architecture synergize to overcome practical challenges. We present a proof-of-concept case study demonstrating the architecture and graph model, discuss the overarching benefits of the architecture, and outline future research directions.

## 2 Materials and methods

The proposed application architecture draws upon the utility of both autoscaling serverless functions and a microservice architecture. Serverless functions are a feature offered by cloud platforms where developers write code that is executed in response to events (like a user interaction), and are automatically scaled up and down by the cloud provider. They are serverless in the sense that developers do not have to worry about server management, and their pay-as-you-go nature makes them cost-efficient for users. Microservice architecture, on the other hand, is a design pattern where an application is structured as a collection of loosely coupled services, which can be developed, deployed, and scaled independently. Anticipating usage patterns of this patient simulation application may be sporadic and synchronized, such as classroom usage leading to surges in demand, the proposed architecture is capable of scaling up and down effectively to meet these needs.

In addition, our proposed architecture considers the challenge of device heterogeneity and limited processing power, especially in the medical education setting. A cross-platform programming language is preferred, which allows developers to write a single codebase that can run on multiple platforms (like Android, iOS, and web), eliminating the need to write different versions of the application for each platform. In this case, React-Native (Masiello and Friedmann, 2017), a popular cross-platform programming language, has been employed.

**FIGURE 1:**
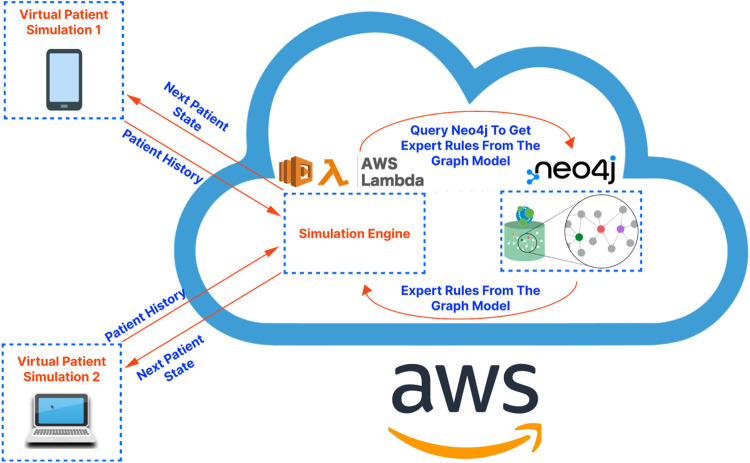
A high-level illustration of the proposed application architecture. The virtual patient simulations on the left-hand side of the diagram represent the front-end application. The cloud on the right-hand side of the diagram represents the cloud services serving as the “back-end” of the application. These services are hosted on Amazon Web Services (AWS) in the demo application/proof-of-concept presented in this article.

For the overall architecture, the cross-platform front-end (written in React-Native) is separated from the back-end simulation engine (running on a serverless function in the cloud) and the graph database (running on a dedicated server in the cloud). This separation, characteristic of microservice-based architectures, has been shown to improve scalability, reliability, and fault tolerance while also facilitating maintenance and debugging tasks (Villamizar et al., 2015). Additionally, serverless functions, due to their autoscaling and developer-friendly nature, enable developers to focus on application logic, leaving resource provisioning and infrastructure management to cloud service providers (Chadha et al., 2022). An illustration of the proposed application architecture is shown in Figure 1. Below we present the details regarding the cross-platform front-end application, the graph database construction, and the simulation engine that drives the patient pathway simulation, respectively.

### 2.1 Front-end application

**FIGURE 2:**
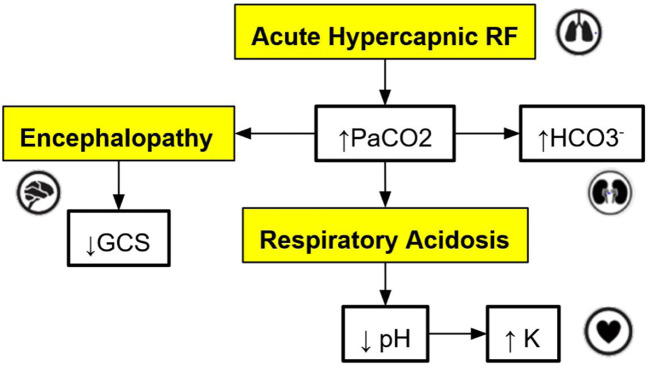
An example of a directed acyclic graph (DAG) depicting a subset of the interactions associated with respiratory acidosis. The boxes with a yellow background are medical concepts, and the boxes with a white background correspond to measurable patient vitals or clinical markers. PaCO2 = partial pressure of carbon dioxide in arterial blood, GCS = Glasgow Coma Scale, HCO $3^{-}$ = Bicarbonate.

The cross-platform front-end application serves as the user interface for trainees and clinicians to interact with the virtual patient simulation by: (a) allowing users to set the initial state of the patient; (b) storing and showing the state of the patient over the course of a simulation; (c) allowing users to select interventions at each step of the simulation as desired; (d) sending the history of patient states to the cloud-hosted simulation engine to obtain the next state of the patient for the next step of the simulation (see Section 2.3 for more details); (e) tracking the relationships, i.e., edges in the graph-model that caused a change in the virtual patient’s state at each step of the simulation; (f) allowing users to connect to the graph database to visualize the relationships defined in the graph model, which influence the trajectory of the state of the virtual patient (see Figure 2 for a sample DAG).

The microservice architecture plays a crucial role here as it does not require embedding complex simulation logic into the front-end application as would be required in a monolithic application design. This division of responsibilities keeps the front-end lightweight and modular, facilitating independent development, better error isolation, and improved overall development speed.

### 2.2 Graph database development

A graph database uses graph structures for semantic queries, with nodes, edges, and properties to represent and store data. This stands in contrast to a traditional SQL or noSQL database which may not natively support relationships between entities. In our study, the graph database is the heart of our simulation application, performing crucial functions like storing the graph model, enabling fast queries, providing visualization tools, and allowing developers to manage the graph model. These graph-database-powered capabilities can assist in maintaining the robustness, flexibility, and scalability of the simulation model.

For this application, the graph models are constructed based on expert rules. Our definition of expert rules takes into account the effects of clinical markers on each other and the causes (like interventions and interactions) that lead to certain effects on organ systems. Using a graph database, the expert rules (defined by clinicians and loaded into Neo4j via CSV files) that drive our simulation can be efficiently queried and updated. A very simple example DAG describing a subset of the interactions of organ systems and biomarkers associated with respiratory acidosis is shown in Figure 2. This DAG is constructed using rules presented in Table 1 (to be elaborated in this section).

**TABLE 1:** The set of expert rules which define the edges in the Neo4j graph shown in Figure 4, and which represent the relationships shown in the DAG in Figure 2. These rules govern the progression of the state of the virtual patient described in the case study in Section 3.

Rule #	Cause/Input	Previous _State_ Of_Cause /Input	New _State_ Of_Cause/Input	Duration	Effected _Clinical _Marker	Impact	P	Time _Until _Effect	Simple_Conditions
1	PaCO2	2	2	30	GCS	−1	0.8	0	
2	PaCO2	1	2	30	GCS	−1	0.8	0	
3	PaCO2	0	2	30	GCS	−1	0.8	0	
4	PaCO2	−1	2	30	GCS	−1	0.8	0	
5	PaCO2	−2	2	30	GCS	−1	0.8	0	
6	PaCO2	1	2	0	pH	−1	1	15	
7	PaCO2	0	1	0	pH	−1	1	15	
8	PaCO2	−1	0	0	pH	−1	1	15	
9	PaCO2	−2	−1	0	pH	−1	1	15	
10	PaCO2	1	2	0	HCO3-	1	0.8	240	
11	PaCO2	0	1	0	HCO3-	1	0.8	240	
12	PaCO2	−1	0	0	HCO3-	1	0.8	240	
13	PaCO2	−2	−1	0	HCO3-	1	0.8	240	
14	pH	2	1	0	K	1	0.8	30	[{Given_Insulin: 0, Duration: 60} {Given_Furosemide: 0, Duration: 60}]
15	pH	1	0	0	K	1	0.8	30	[{Given_Insulin: 0, Duration: 60} {Given_Furosemide: 0, Duration: 60}]
16	pH	0	−1	0	K	1	0.8	30	[{Given_Insulin: 0, Duration: 60} {Given_Furosemide: 0, Duration: 60}]
17	pH	−1	−2	0	K	1	0.8	30	[{Given_Insulin: 0, Duration: 60} {Given_Furosemide: 0, Duration: 60}]

Note that the simple DAG depicted in Figure 2 could be a part of a much larger DAG with many more medical concepts, measurable patient vitals, organ systems, and relationships (Lal et al., 2020b). Representing the causal pathways within the human body in an intuitive way is particularly important in a clinical setting as information overload has been correlated with an increase in medical errors (Pickering et al., 2010). Accordingly, DAGs have been utilized by clinicians in recent work to model the complex underlying causal pathways that drive the trajectory of a patient in an intuitive and visualizable way (Lal et al., 2020a). In particular, DAGs can be used to effectively model complex causal pathways within the human body as they provide a natural way to model high-dimensional directed relationships. From a simulation development perspective, instead of needing to define each new simulation scenario by hand, utilizing a graph-based simulation engine allows the number of supported scenarios to grow naturally over time as new patient vitals, clinical markers, interventions, and their associated interactions (edges) are added to the graph over the course of the iterative expert rule refinement and validation process.

The graph database utilized in this work is Neo4j (Neo4j Graph Data Platform, 2021), which has been shown to be effective at storing, querying, and analyzing graph data such as knowledge graphs (Chen, 2022). Other graph databases are also available including Amazon Neptune (Amazon Web Services, 2024) and TigerGraph (TigerGraph, 2023), among others. When developing rules for the graph model stored in the Neo4j graph database, we first define independent expert rules that have been agreed upon by the experts in the field through a formal consensus process (Gary et al., 2022). Table 1 contains sample rules expressed in the spreadsheet format to help illustrate the rule structure that is compatible with the Neo4j data structure. In the patient simulation, each rule is activated by a single triggering clinical marker or intervention (the “Cause/Input” column of the spreadsheet), and each rule causes a new incremental change or an absolute change in a single impacted clinical marker (the “Effected_Clinical_Marker” column of the spreadsheet) when all conditions for the expert rule are satisfied. Currently, states of the clinical markers are represented as integer variables (−2,-1,0,1,2) and can be color-coded in the front-end user interface. The integer values map to different value ranges of measurable biomarkers. For example, level 2 for PaCO2 corresponds to values between 71 and 120 mmHg. In the front-end application, a number randomly drawn within this range will be displayed to users, providing users with an experience closer to their regular interactions with electronic health records.

The first rule in Table 1 says, when the patient’s PaCO2 level stays at a high level (2) for a duration of 30 min, then GCS (Glasgow Coma Scale) decreases by 1 level with a probability of 0.8. In this example, PaCO2 is the “Cause/Input” of the rule, GCS is the “Effected_Clinical_Marker”, 0.8 is the “Probability”, −1 is the “Impact”, and 0 is the “Time_Until_Effect” (in minutes). The columns “Previous_State_Of_Cause/Input” and “New_State_Of_Cause/Input” describe what needs to happen to the value of the “Cause/Input” for the rule to be triggered. There are three possible triggers that we can account for: The “Cause/Input” increases, decreases, or stays at a particular value over the specified “Duration”. In this example, the “Previous_State_Of_Cause/Input” and the “New_State_Of_Cause/Input” of PaCO2 are both high (level 2), and the “Duration” is 30 min meaning that this rule is triggered after PaCO2 has been at level 2 for 30 min. By specifying a “Duration”, we can have different rules for changes that occur acutely/quickly, or which occur slowly over time. We can also model rules such as “IF PaCO2 is > 70 mmHg (FOR 30 min) THEN GCS decreases” which requires that a particular “Cause/Input” (PaCO2 in this case) stays at a particular value (in this case, at a high value) for some duration. Note that, by allowing for capturing the “Duration”, the simulation is no longer memoryless and the applicability of a rule is based on the historical patient trajectory.

The effect of each rule on the impacted clinical marker is stored in the “Impact” column and is represented by one of the following integers: (−2,-1,1,2). The negative (positive, resp.) integers represent a decrease (an increase, resp.) in the value or level of the impacted clinical marker. In this example (rule #1), the GCS level will be decreased by 1 level, from its current level, and the time-lapse it needs to be effective is stored in the “Time_Until_Effect” column (with zero meaning being effective immediately in this case). To handle cases where multiple rules are simultaneously applying changes to a single clinical marker during one step of the simulation, we introduce two types of rules, one causes an incremental change, meaning that its effect is additive to others that are also incremental. The other type is “absolute”, which will override other rules once applied. In this simple example, all rules cause incremental changes.

For a rule to be activated, relevant conditions defined in the rule must be satisfied. The simple conditions are one or more independent conditions that all must be satisfied for a rule to take effect. Rules 14–16 in Table 1 have two simple conditions, {Given_Insulin: 0, Duration: 60} and {Given_Furosemide: 0, Duration: 60}. These conditions mean that rules 14–16 will only be applied if the patient has not been given Insulin or Furosemide during the last 60 min.

Meanwhile, complex conditions are the conditions that are satisfied if at least one of a possible set of conditions is satisfied. For example, a complex condition expressed as “[{ Brain_Swelling: 0, Duration: 0 },{ Mannitol: 1, Duration: 30 }]” requires that at least one of the following must be true: (a) the patient must have no current brain swelling (b) they must have received Mannitol 30 min ago.

If all of the conditions for a rule are satisfied, we then apply the rule with the probability listed in the “P” column. The probability characterizes the chance that a certain change in the human body will occur to maintain a level of stochasticity in the simulation model.

This precise structure for expressing expert rules allows us to capture the majority of the common rules using a systematic format that is interoperable with graph databases, and enables us to customize each expert rule based on the applicability of each property.

### 2.3 Cloud-hosted simulation engine

The cloud-hosted simulation engine is responsible for executing the simulation according to the graph model stored in the database and the user interactions captured by the front-end application. The engine runs on a serverless function (on a Function as a Service platform, like Amazon Web Services Lambda or Google Cloud Functions), allowing it to scale seamlessly in response to demand. These serverless computing platforms provide developers with a high degree of flexibility and scalability, as they only need to be concerned with application code and can leave infrastructure management to the service provider.

The engine is designed to take the current state of the patient, as well as any user actions (like giving a medication or performing a procedure), and calculate the resulting state of the patient. For this, it queries the graph database for relevant rules, performs calculations, and sends the new patient state back to the front-end application. As a benefit, the engine does not have to store any state itself, making it inherently scalable and resilient. Also, being decoupled from the front-end and the database, it can be independently developed, tested, and deployed, which reduces the complexity of the overall system.

All current and future rules can be processed in a uniform way using the same code (the code running in the simulation engine as shown in Figure 1). This means that rules in the graph database can be added and updated in the future independently without the need for the developers to write any new code. Specifically, to obtain the next patient state at each step in the simulation, the front-end application sends the complete patient history to the simulation engine and waits for a response which includes:

1. The next state (described by the states of all clinical markers) of the patient.
2. The rules that were applied (if any) which impacted the next state of the patient.

The upper and lower limits for the value of each clinical marker (currently some appropriate range between “very low” (−2) and “very high” (2)) and the lower and upper bound for each intervention (between “no intervention” (0) and “high dose intervention” (2)) are defined in the simulation engine and enforced at each step. Similarly, the length between each step in the simulation is defined (currently “15 min”).

**FIGURE 3:**
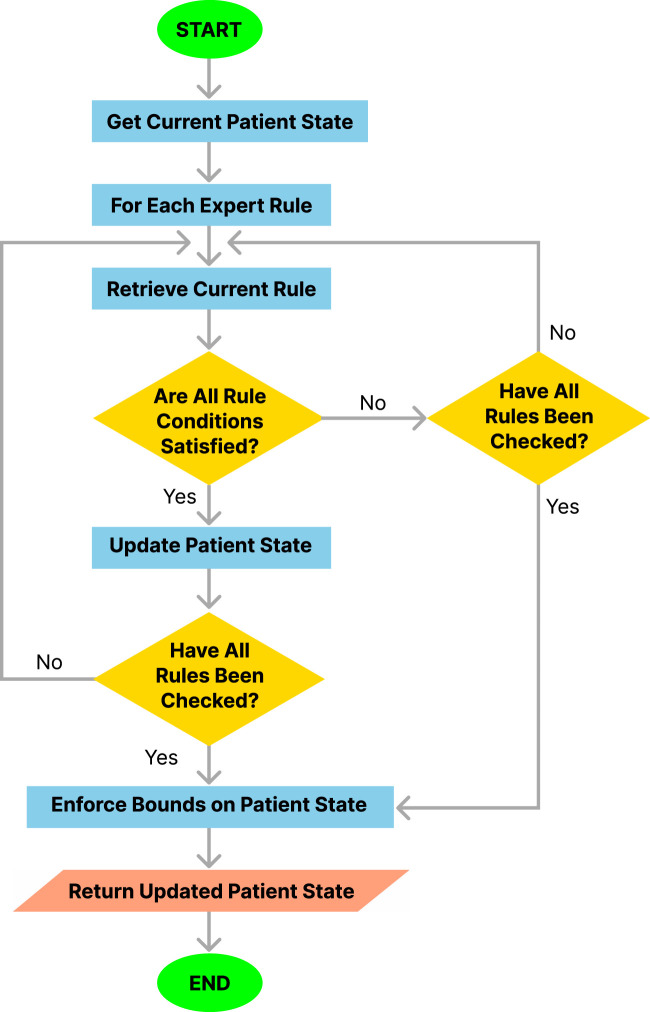
Flowchart of the simulation engine algorithm.

The procedure followed by the simulation engine at each step of the simulation is outlined in Algorithm 1 and illustrated in Figure 3. This procedure integrates several functions in a modular approach to rule application and state updates.

Algorithm 1 Simulation Engine Overarching Algorithm.

**Require:** Time_Between_Steps=15

**Require:** t=0,1,…,T ⊳ The steps of the simulation, each of which is Time_Between_Steps minutes apart
**Require:** $Variable\text{\_}\mathrm{Names}=\left\{{Name}_{1},{Name}_{2},\ldots ,{Name}_{n}\right\}$

**Require:** $Lower\text{\_}\mathrm{Bounds}=\left\{l_{1},l_{2},\ldots ,l_{n}\right\}$

**Require:** $Upper\text{\_}\mathrm{Bounds}=\left\{u_{1},u_{2},\ldots ,u_{n}\right\}$

**Require:** $Patient\text{\_}\mathrm{History}=\left\{h_{0},h_{1},h_{2},\ldots ,h_{t}\right\}$

**Require:** $Expert\text{\_}\mathrm{Rules}\leftarrow \left\{{Rule}_{1},{Rule}_{2},\ldots ,{Rule}_{m}\right\}$

 1: $h_{t+1}=h_{t}$

 2: InitializeSimulation ( Time_Between_Steps , t , Variable_Names , Lower_Bounds , Upper_Bounds , Patient_History , Expert_Rules )
 3: **for** j=1 to m **do**
 4: Current_Rule=Expert_Rules[j]

 5:   ApplyRules ( Current_Rule , Patient_History , $h_{t+1}$ , Time_Between_Steps )
 6: **end for**
 7: **for** Var in Variable_Names **do**
 8:   EnforceBounds ( $h_{t+1}$ , Var , Lower_Bounds , Upper_Bounds )
 9: **end for return** $h_{t+1}$

#### 2.3.1 InitializeSimulation function

The InitializeSimulation procedure initializes the parameters and patient history required for the simulation. It ensures that all necessary data is correctly set up before the main simulation steps begin.

Algorithm 2 InitializeSimulation Procedure.

1: **procedure** InitializeSimulation ( Time_Between_Steps , t , Variable_Names , Lower_Bounds ,
 
Upper_Bounds , Patient_History , Expert_Rules )
2:  Initialize parameters and patient history
3: **end procedure**

#### 2.3.2 ApplyRules function

The ApplyRules function applies the relevant rules from the expert rules set to update the patient’s state. It checks if the conditions for each rule are met and, if so, updates the patient state accordingly.

Algorithm 3 ApplyRules Function.

1: **function** ApplyRules ( Current_Rule , Patient_History , $h_{t+1}$ , Time_Between_Steps )
2: $Duration\text{\_}\mathrm{Steps}=\frac{Current\text{\_}\mathrm{Rule}\left[Duration\right]}{Time\text{\_}Between\text{\_}Steps}$

3: $Index\text{\_}Of\text{\_}Newest\text{\_}Measurement\text{\_}To\text{\_}Look\text{\_}At=\frac{Current\text{\_}\mathrm{Rule}\left[Time\text{\_}Until\text{\_}E\mathrm{ff}ect\right]}{Time\text{\_}Between\text{\_}Steps}$

4: Index_Of_Oldest_Measurement_To_Look_At=Index_Of_Newest_Measurement_To_Look_At+

   
Duration_Steps+1

5:  **if** Index_Of_Oldest_Measurement_To_Look_At>t **then**
6:   **return False**
7:  **end if**
8: Cause=Current_Rule[Cause/Input]

9:  **if** $h_{t-Index\text{\_}Of\text{\_}Oldest\text{\_}Measurement\text{\_}To\text{\_}Look\text{\_}At}\left[Cause\right]\neq Current\text{\_}\mathrm{Rule}\left[Previous\text{\_}State\text{\_}Of\text{\_}Cause/Input\right]$

  **then**
10:   **return False**
11:  **end if**
12:  **end if** $h_{t-Index\text{\_}Of\text{\_}Newest\text{\_}Measurement\text{\_}To\text{\_}Look\text{\_}At}\left[Cause\right]\neq Current\text{\_}\mathrm{Rule}\left[New\text{\_}State\text{\_}Of\text{\_}Cause/Input\right]$ **then**
13:   **return False**
14:  **end if**
15: MaxValue=max(Current_Rule[Previous_State_Of_Cause/Input] ,
  
Current_Rule[New_State_Of_Cause/Input])

16: MinValue=min(Current_Rule[Previous_State_Of_Cause/Input] ,
  
Current_Rule[New_State_Of_Cause/Input])

17:  **for** k=(t−Index_Of_Oldest_Measurement_To_Look_At+1) to
  
(t−Index_Of_Newest_Measurement_To_Look_At−1) **do**
18:   **if** $h_{k}\left[Cause\right]>MaxValue\text{or}h_{k}\left[Cause\right]<MinValue$ **then**
19:    **return False**
20:   **end if**
21:  **end for**
22:  **if** HandleConditions ( h,Current_Rule,Index_Of_Newest_Measurement_To_Look_At ,
  
Time_Between_Steps ) **then**
23:   UpdatePatientState ( Current_Rule , $h_{t+1}$ )
24:   **return True**
25:  **else**
26:   **return False**
27:  **end if**
28: **end function**

#### 2.3.3 HandleConditions function

The HandleConditions function evaluates whether the conditions for applying a rule are satisfied based on the patient’s history and the specifics of the rule. It checks whether the current rule contains a simple condition or a complex condition and whether these are satisfied over the most recent steps to be analyzed prior to moving to the next time instance. We added simple and complex conditions during the rule construction process to ensure that the expert rules are capable of fully capturing the intricate relationships between organ systems in the human body. For example, the administration of propofol to a critically ill patient should result in a drop in GCS as well as a drop in MAP. However, if phenylephrine was administrated at the same time as propofol, a drop in MAP would have not occurred. Then, administration of phenylephrine would be included in the simple condition of the rules denoted as { Given_Phenylephrine : 0} suggesting that phenylephrine should not be currently effective for this rule to be applicable.

The algorithm returns a Boolean variable ConstraintsSatisfied being “True” if all constraints are satisfied, and “False” otherwise. The condition check operation shares a similar structure as the main algorithm, e.g., screening the states and managing the time indexes, and the details are skipped for the interest of space.

Algorithm 4 HandleConditions Function.

1: **function** HandleConditions ( h,Current_Rule,Index_Of_Newest_Measurement_To_Look_At, Time_Between_Steps )
2:  Evaluate simple and complex conditions of the rule
3:  **return** all conditions are satisfied **and also** rand(Unif(0,1))≤Current_Rule[Probability]

4: **end function**

#### 2.3.4 UpdatePatientState function

The UpdatePatientState procedure applies the impacts of a rule to the patient’s state if the conditions for that rule are met.

Algorithm 5 UpdatePatientState Procedure.

1: **procedure** UpdatePatientState ( Current_Rule , $h_{t+1}$ )
2: $h_{t+1}\left[E\mathrm{ff}ected\text{\_}Clinical\text{\_}Marker\right]+=Current\text{\_}\mathrm{Rule}\left[Impact\right]$

3: **end procedure**

#### 2.3.5 EnforceBounds function

The EnforceBounds procedure ensures that the values of all clinical markers and interventions remain within their predefined bounds (e.g., when incremental rules are applied, check if the values go beyond −2 or +2 ). If a value exceeds its bounds, it is set to the respective limit.

Algorithm 6 EnforceBounds Procedure.

1: **procedure** EnforceBounds ( $h_{t+1}$ , Var , Lower_Bounds , Upper_Bounds )
2:  **if** $h_{t+1}\left[Var\right]<Lower\text{\_}\mathrm{Bounds}\left[Var\right]$ **then**
3: $h_{t+1}\left[Var\right]=Lower\text{\_}\mathrm{Bounds}\left[Var\right]$

4:  **else if** $h_{t+1}\left[Var\right]>Upper\text{\_}\mathrm{Bounds}\left[Var\right]$ **then**
5: $h_{t+1}\left[Var\right]=Upper\text{\_}\mathrm{Bounds}\left[Var\right]$

6:  **end if**
7: **end procedure**

The algorithmic approach modularizes the process into distinct functions, each responsible for specific aspects of the simulation, thus enhancing clarity and maintainability. The overarching algorithm (Algorithm 1) orchestrates the workflow, ensuring that all necessary steps are performed in sequence, while the individual functions handle initialization, rule application, condition checking, patient state updating, and enforcing bounds.

To summarize, the simulation engine runs on a serverless function in the cloud and performs the following functions: (a) receives the history of a virtual patient from a user’s front-end application; (b) calculates the next state of the virtual patient for the next step of the simulation by analyzing the history of past states of the virtual patient, querying the graph database to obtain the relevant relationships from the graph-model which may cause a change in the state of the patient, and applying the queried relationships as appropriate to calculate the next state of the patient; (c) returns any rules that were applied and the next state of the virtual patient for the next step of the simulation to the user’s front-end application.

## 3 Results

**FIGURE 4:**
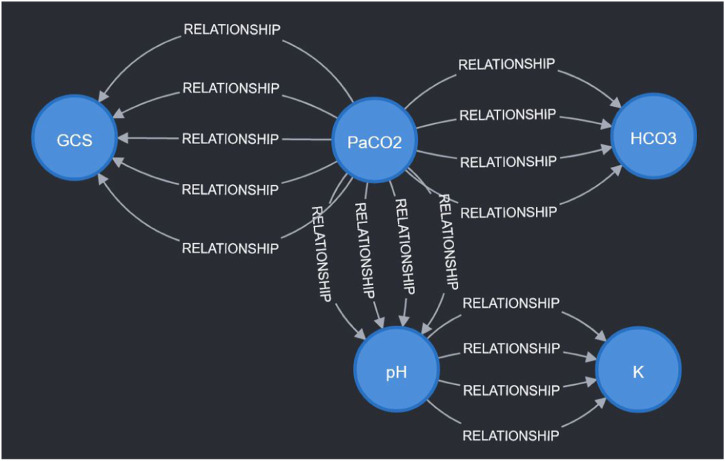
Visualization of sample expert rules stored in the Neo4j graph database. Each node in the graph corresponds to a measurable vital or clinical marker in Figure 2. Each directed edge corresponds to a specific expert rule in Table 1. The detailed cause-effect will be displayed when the specific “relationship” edge is clicked in the Neo4j workspace.

To demonstrate the viability of the proposed simulation architecture, we will walk through a short case study that considers a virtual patient whose state is defined in terms of the five clinical markers shown in the DAG in Figure 2 and the corresponding nodes in the Neo4j graph in Figure 4. The trajectory of the patient will be determined by the set of edges shown in the Neo4j graph in Figure 4, each of which corresponds to an expert rule defined in Table 1. The trajectory of the patient’s state throughout this case study is summarized in Table 2, and the rules from Table 1 that were applied at each step of the simulation (each step is 15 min) are described in the “Applied Rules” column of Table 2.

**TABLE 2:** The patient’s state throughout Section 3 case study.

Time (min)	PaCO2	pH	HCO3-	GCS	K	Applied rules
0	1	0	0	0	0	
15	2	0	0	0	0	
30	2	−1	0	0	0	6
45	2	−1	0	0	0	
60	2	−1	0	0	1	16
75	2	−1	0	−1	1	1
90	2	−1	0	−2	1	1
105	2	−1	0	−2	1	
120	2	−1	0	−2	1	

This case study (respiratory acidosis) is crafted to allow for a manual prospective validation to assist in a quick understanding of the simulation mechanism. In the real implementation, the user will first choose a clinical scenario (e.g., chronic obstructive pulmonary disease exacerbation, or sepsis), along with the most relevant clinical markers and the corresponding rules related to this clinical scenario will be identified. Each clinical scenario is typically associated with dozens of clinical markers and rules, e.g., 70 rules for a demonstration version for validation in a related study (Rovati et al., 2024).

### 3.1 Initializing the simulation

To initialize the simulation, we first need to set the lower and upper bounds for each vital/clinical marker that we have. In this case study, the simulation engine was configured to use the upper and lower bounds: $Lower\text{\_}Bounds=\left\{PaCO2:-2,pH:-2,HCO3^{-}:-2,GCS:-2,K:-2\right\}$ , $Upper\text{\_}Bounds=\left\{PaCO2:2,pH:2,HCO3^{-}:2,GCS:0,K:2\right\}$ .

Also, we need to define an initial $Patient\text{\_}History=\left\{h_{0},h_{1}\right\}$ for the patient. Let us assume that at the first step of the simulation, step t=0 (row 1 of Table 2), the patient had a slightly elevated level of PaCO2 (denoted by a value of “1”) and a normal level of all the other clinical markers (denoted by a value of “0”). Then, 15 min later at step t=1 (row 2 of Table 2), the patient had a very elevated PaCO2 level (denoted by a value of “2”), but still had a normal level (level “0”) for all the other clinical markers. In this case, the Patient_History described in Algorithm 1 is initialized as $h_{0}=\left\{PaCO2:1,pH:0,HCO3^{-}:0,GCS:0,K:0\right\}$ and $h_{1}=\left\{PaCO2:2,pH:0,HCO3^{-}:0,GCS:0,K:0\right\}$ .

### 3.2 The patient’s state trajectory during the simulation

As shown in Table 2, the first rule applied is Rule # 6 at time t=30 minutes. This is expected as Rule # 6 is triggered by an increase in PaCO2 from a slightly elevated level (a value of “1”) to a very elevated level (a value of “2”). Since the duration is 0 min for this rule, this rule is triggered as soon as the value of PaCO2 changes from “1” to “2”. However, this rule has a delayed “Time_Until_Effect” of 15 min which means that the “Impact” of the rule is applied 15 min after the rule is triggered. Therefore, since the rule was applied at time t=30 minutes, the rule was triggered 15 min earlier, at time t=15 minutes. Once the rule was triggered it was guaranteed to be applied since the rule’s probability, P , is 100%.

Next, at time t=60 Rule #16 was applied. Rule #16 is triggered by a decrease in pH from a normal level (level “0”) to a slightly low level (level “-1”). After the decrease occurs, this rule is delayed by a “Time_Until_Effect” of 30 min. Therefore, the change in pH must have occurred 40 min earlier, which we can see occurred in Table 2 as pH decreased from normal (level “0”) at time t=15 to slightly low (level “-1”) at time t=30 . It is therefore in alignment with our expectations that Rule #16 is applied 30 min later at time t=60 minutes due to the rule’s “Time_Until_Effect” of 30 min.

At time t=75 one rule was applied, Rule #1. Rule # 1 is triggered by PaCO2 being at level “2” for 30 min, and looking at the patient’s state history in Table 2, we can see that at time t=75 minutes, the patient had actually already had a PaCO2 level of “2” for 60 min. Since this rule has a “Time_Until_Effect” of 0 min, we know that once this rule is triggered, its “Impact” is instantly applied. Subtracting the rule’s “Duration” of 30 min from the 60 min that the patient’s PaCO2 level was “2”, we can see that starting at time t=30 minutes the rule was being triggered. However, as indicated by column P of Table 1, Rule #1 only has an 80% probability of being applied each time it is triggered. This means that the rule was only applied on the third time that it was triggered (the 20% chance that the rule would not be applied hit the first two times it was triggered, at t=45 and t=60 ).

At time t=90 , Rule #1 was applied again, further decreasing GCS to its lower bound of “-2”. As we can see, Rule #1 was not decreased at time t=105 or t=120 even though Rule #1 was still being triggered since GCS can not decrease below its lower bound (below a value of “-2”).

In conclusion, we can see that the trajectory of the patient’s state throughout the case study (Table 2) is in alignment with our expectations based on our expert rules (Table 1).

## 4 Discussion

The presented work introduces an application architecture designed to overcome various challenges inherent in the dynamic realm of healthcare simulations. Specifically, it is constructed to seamlessly scale to accommodate a growing user base with sporadic and correlated usage patterns, making it universally accessible across a multitude of platforms. It is also built to operate reliably under various conditions while ensuring fault-tolerance and easy maintainability.

A key aspect of this architecture is that it does not question the validity of expert rules, but rather focuses on the execution of these rules within the simulation. Therefore, during the validation phase, an unexpected simulation behavior due to an incorrect expert rule or its faulty implementation can be handled separately. For instance, if an erroneous simulation result is due to an incorrect expert rule, the developer only needs to update the graph database without touching the simulation engine. This will also improve the handling of the changes in the clinical management of patients in the intensive care unit where the scientific premise and the interventions change according to an evolving body of evidence.

Because of the stochastic nature of the simulation and the scale of the model, it is infeasible to validate the model based on specific values of each individual clinical marker realized in each simulation run. Rather, we focus on the clinical trajectory and examine whether the trajectory over an initial 6-h span from the time of admission is concordant with the expectation (e.g., samples from real patient trajectories or crafted virtual patients with the same clinical scenario). Our commitment to enhancing the validity and utility of this simulation application extends beyond the present study. We understand the importance of rigorous evaluation and ablation studies and are actively engaged in further research to refine and validate the expert rules that underpin the simulation. We are employing rigorous methodologies to calibrate the decision-making algorithms based on real-world patient data and physician inputs. To ascertain the application’s effectiveness as an educational tool and its ability to satisfy user requirements, we have initiated a mixed-methods study involving first-year Internal Medicine residents (Gary et al., 2023; Rovati et al., 2024). These user testing sessions are specifically designed to assess the usability of the application, the workload it presents to users, the usability of the application, and the satisfaction of learners. We anticipate that the findings from these sessions will provide invaluable insights and guide iterative refinement of the application design to better cater to user needs.

Looking ahead, there are numerous avenues for enhancing the proposed architecture’s scalability, reliability, efficiency, and performance. Such improvements are crucial for realizing high-fidelity graph-based simulation models capable of functioning as decision support tools for clinicians at the bedside. Our vision is to use these models as digital twins and interpretable counterparts to less transparent associative AI models, facilitating patient diagnosis and optimal treatment prediction in real-time settings (see, for example, (Komorowski et al., 2018; Chakshu and Nithiarasu, 2022; Sun et al., 2022)). The interpretability aspect is particularly crucial in healthcare, given the reluctance among clinicians to adopt “black-box” AI models (Dang et al., 2021; Lal et al., 2022).

Specifically, to utilize a data-driven approach to further validate the patient simulation application, it is necessary to extract meaningful data points from the current plethora of variables thereby improving the signal-to-noise ratio. This approach would involve the current electronic health record data being mapped to experimentally proven physiological concepts (e.g., utilizing our approach with DAGs and validated expert rules). The future iterations of this scalable patient simulation application will also include a “plug-in” feature with the current electronic health record, which will seamlessly integrate the real-time data and interoperability of the proposed virtual testing environment with the current clinical infrastructure for medical education, *in silico* research, and clinical decision support.

To realize these visions, an exciting future direction involves the utilization of graph algorithms like Graph Neural Networks for link prediction. This would improve the accuracy of the graph model that drives the virtual patient simulation. Graph Neural Networks have demonstrated state-of-the-art results in predicting synthetic lethality and drug-target interaction in biomedical networks (Long et al., 2022). Therefore, applying these algorithms to a graph model based on DAGs, illustrating causal relationships and intricate pathophysiological interactions within the human body, could potentially yield impressive results.

Another intriguing prospect is to enhance the efficiency of querying the Neo4j graph database. Currently, the simulation engine examines all rules upon querying the graph database, even those that do not meet the application conditions. Future work should aim to develop more specific queries using Neo4j’s cypher query language. This could traverse only nodes or edges of a specific type or with particular properties, increasing query efficiency. However, this requires careful reconsideration of how the data is structured within the database, given the unique set of simple and complex conditions associated with each rule.

Lastly, the incorporation of parallel computing within the cloud-hosted simulation engine could significantly boost its performance. Recent research has shown that integrating parallel computing within serverless functions drastically enhances performance and reduces costs (Kiener et al., 2021). Future studies could adapt these findings to elevate the performance of our simulation engine. These initiatives, when realized, could greatly advance the capabilities of the proposed architecture, moving us closer to our ultimate goal of creating a robust and scalable tool for healthcare simulations.

## Data Availability

The data analyzed in this study is subject to the following licenses/restrictions: The datasets used for this study are not publicly available. Requests to access these datasets should be directed to YD, dong.yue@mayo.edu.
